# Substrate-Sequence Effects on Pollutant Removal and Microbial Succession in Modular Constructed Wetlands Under Plateau Low-Temperature Habitat Conditions

**DOI:** 10.3390/microorganisms14071549

**Published:** 2026-07-15

**Authors:** Yansong Wang, Renxu Wang, Yongchen Zong, Xiangyu Chen

**Affiliations:** 1 Research Center of Civil, Hydraulic and Power Engineering of Xizang, Department of Education of Xizang Autonomous Region, Xizang Agricultural and Animal Husbandry University, Linzhi 860000, China; wys1844974834@163.com; 2 The College of Water Conservancy and Civil Engineering, Xizang Agricultural and Animal Husbandry University, No. 100 Yucai West Road, Bayi District, Linzhi 860000, China; 15611759752@163.com (R.W.); chenxiangyu@xza.edu.cn (X.C.)

**Keywords:** domestic wastewater, modular constructed wetland, microbial succession, low C/N ratio, substrate configuration

## Abstract

Constructed wetlands operated in plateau habitats may experience constrained biological treatment because low temperature, low atmospheric pressure, and low-carbon wastewater can jointly limit microbial metabolism. This 80-day pilot screening study evaluated three nonreplicated modular constructed wetland configurations (MCW1-MCW3) containing different sequences of zeolite, ceramsite, and quartz sand and planted with *Veronica anagallis-aquatica*. Each configuration consisted of one independent treatment train; therefore, the comparisons were interpreted as configuration-specific and exploratory rather than as statistically generalizable treatment effects. Pollutant-removal performance and microbial community succession were evaluated through repeated water-quality monitoring and 16S rRNA gene sequencing. MCW1 showed the highest observed mean NH_4_^+^-N removal efficiency (88.6%), whereas MCW3 showed the highest observed mean TP and COD removal efficiencies (79.56% and 47.40%, respectively) and an NH_4_^+^-N removal efficiency of 85.51%. TN removal by MCW3 remained limited at 20.49%, consistent with carbon limitation of denitrification. Under the naturally low-temperature plateau laboratory conditions, the observed COD reduction indicated partial mineralization or retention of organic pollution loads, potentially supported by substrate biofilms and cold-adapted microbial assemblages. Apparent module-contribution analysis suggested that zeolite contributed substantially to NH_4_^+^-N reduction, whereas ceramsite contributed to TP and COD removal under the tested sequences. Because plant biomass and tissue nutrient contents were not measured, nitrogen and phosphorus removal could not be attributed quantitatively to hydrophyte uptake. Overall, substrate sequence influenced pollutant-removal patterns and microbial community assembly, providing preliminary evidence for habitat-adapted optimization of modular constructed wetlands for plateau domestic wastewater.

## 1. Introduction

With the continuous social and economic development, environmental protection has attracted increasing global attention. Water pollution is a major global environmental challenge that threatens water security and aquatic ecosystem functioning [1]. Constructed wetlands (CWs) are cost-effective and sustainable nature-based wastewater-treatment systems that emulate natural wetland processes and have been implemented in more than 50 countries [2,3]. They achieve pollutant removal through the synergistic effects of substrates, plants, and microorganisms, and have been widely applied in many fields. Substrates contribute to pollutant removal through filtration, interception, adsorption, and ion exchange while also providing physical support for plants and attachment surfaces for microorganisms [4]. Wetland plants facilitate pollutant removal through nutrient uptake, root-mediated oxygen release, enhanced hydraulic retention, and the provision of surfaces for biofilm development and microbial attachment [5]. Microorganisms are major biological drivers of pollutant removal in constructed wetlands through organic-matter mineralization, nutrient transformation, biodegradation, biosorption, and biomineralization [6].

The Qinghai–Tibet Plateau, the world’s largest and highest plateau, contains numerous lakes and extensive frozen-water reserves and is therefore often referred to as the ‘Asian Water Tower’ [7,8]. Its water resources support the domestic and industrial water needs of nearly 2 billion people. Xizang Autonomous Region is characterized by a vast territory, sparse population, and widely dispersed rural settlements. In many remote villages, constructing centralized wastewater treatment plants and extensive sewer networks is technically difficult and economically inefficient because of the long transport distances, complex terrain, limited energy supply, and shortage of specialized maintenance personnel. Constructed wetlands therefore represent a potentially suitable decentralized alternative, as they can be installed near wastewater sources, operated with relatively low energy input, and maintained with limited technical support. In this study, the plateau setting is treated as a habitat context rather than as a broad geographic claim. The experiment was conducted for 80 days in a laboratory in the Xizang Autonomous Region at approximately 3000 m above sea level, without supplementary lighting or active temperature control. Thus, the systems were exposed to naturally varying indoor light and low-temperature conditions that reflected a high-altitude operating environment. These conditions are central to the interpretation of the results because microbial mineralization of organic matter, nitrification, and denitrification are all temperature-sensitive processes. Constructed wetlands (CWs) operated on the plateau are also exposed to hypoxia, intense solar radiation, and low atmospheric pressure, while the wastewater to be treated often has a low carbon-to-nitrogen (C/N) ratio. These conditions create two simultaneous bottlenecks. First, low temperatures suppress microbial growth and metabolic activity, slowing the degradation and transformation of pollutants [9]. Microbial richness and abundance in CWs are consequently higher in summer and autumn than in winter [10]. Second, insufficient organic carbon limits complete denitrification and reduces nitrogen removal from low-C/N wastewater [11]. A conventional single-stage CW is therefore unlikely to maintain stable performance when temperature-limited microbial kinetics and carbon-limited denitrification occur simultaneously. Modularization addresses this mismatch by separating treatment functions into replaceable units, creating sequential microenvironments, and allowing future adjustment of hydraulic retention, aeration, or carbon supplementation according to local plateau conditions.

Despite their advantages, conventional constructed wetlands (CWs) remain constrained by substrate clogging. Solids generated through suspended-matter deposition and chemical precipitation, together with biofilm accumulation and root residues, progressively reduce pore space, hydraulic conductivity, treatment capacity, and service life [12]. Modular designs have therefore been explored to permit selective substrate replacement and assign different treatment functions to individual units. Chen et al. [13] identified the No. 4 modular aggregate substrate as the best-performing medium for ammonium nitrogen adsorption, achieving removal efficiencies of 69.1% and 68.5% under the two tested conditions. Liu et al. [14] found that CW1 favored ammonium nitrogen removal, whereas CW2 performed better for total phosphorus and chemical oxygen demand; modular substrate replacement also reduced costs by 40%. Taken together, these studies demonstrate the engineering value of replaceable media and function-specific configurations, but they largely evaluate individual substrate combinations or pollutant-removal outcomes under short-term or ambient-temperature conditions. Recent studies likewise emphasize construction flexibility [15] or pollutant-removal performance, with laboratory tests conducted at 18–23 °C [16]. This evidence does not establish whether such modules retain their hydraulic and microbial functions under the combined low temperature, hypoxia, intense solar radiation, and carbon limitation of the Qinghai–Tibet Plateau. Cold-climate performance remains an acknowledged knowledge gap, particularly for nitrogen removal and microbial degradation [17]. The key unresolved questions are therefore how plateau conditions alter clogging development and functional microbial succession, how these changes affect nitrification and denitrification across modules, and whether module-specific substrate replacement can maintain treatment stability and economic advantages during long-term operation. Addressing these questions is necessary to move modular CWs from pollutant-specific optimization toward habitat-adapted treatment systems for high-altitude regions.

To address these knowledge gaps, we conducted an 80-day pilot study in a plateau laboratory at an altitude of approximately 3000 m. Three modular constructed wetland (MCW) systems were tested as nonreplicated substrate-sequence configurations rather than as true experimental replicates. Each system comprised three acrylic modules (33 cm × 20 cm × 23 cm; nominal volume, 15.18 L per module and 45.54 L per system), and each module contained 10 kg of zeolite, ceramsite, or quartz sand. The order of the three substrates differed among MCW1, MCW2, and MCW3. Each MCW system had a nominal geometric volume of 45.54 L and a calculated hydraulic working volume of 40 L. The three systems shared an influent tank, and each system was operated independently at a design flow rate of 0.02 m^3^ d^−1^ and a hydraulic retention time of 2 d. Two questions guided the study. First, how did the three substrate sequences differ descriptively in their removal of NH_4_^+^-N, TN, TP, and COD under plateau low-temperature habitat conditions? Second, were the observed performance patterns accompanied by differences in microbial community succession across substrates and modules? We hypothesized that substrate order would create distinct adsorption properties and redox microenvironments, resulting in pollutant-specific removal patterns. We further expected systems enriched in nitrifying, denitrifying, and organic-matter-degrading microorganisms to show more favorable nitrogen and COD removal. This was a screening study, not a replicated full-scale validation. Its conclusions are therefore limited to substrate-sequence performance and microbial responses during 80 d under the tested plateau laboratory conditions.

## 2. Materials and Methods

### 2.1. Experimental Design

**Figure 1:**
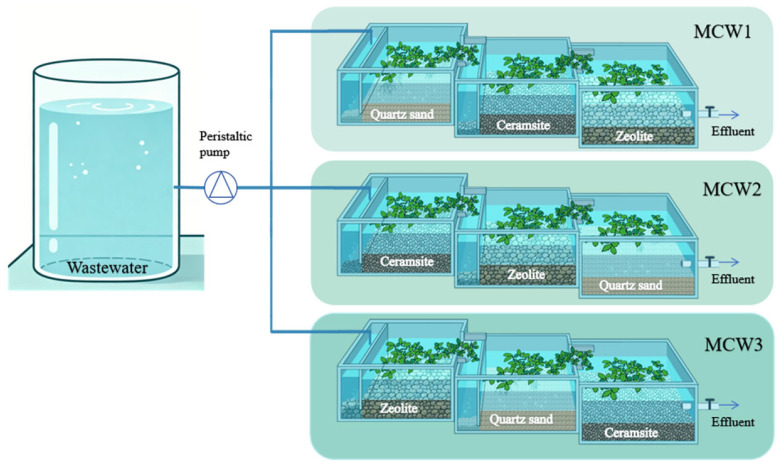
Schematic diagram of the experimental setup.

The experimental setup is shown in Figure 1, which was placed beside the south-facing window in a laboratory at an altitude of 3000 m. The meteorological conditions during the experimental period (June–August 2025) in Bayi District, Linzhi are summarized as follows. In June, the monthly mean daily maximum and minimum temperatures were 11 °C to 22 °C, with an extreme minimum temperature of 6 °C. In July, the monthly mean daily maximum and minimum temperatures were 15 °C to 28 °C, and the extreme minimum temperature was 8 °C. In August, the monthly mean daily maximum and minimum temperatures were 17 °C to 29 °C, with an extreme minimum temperature of 12 °C. This experiment was operated under indoor natural light conditions. No supplementary lighting, light-shielding facilities, or temperature control devices were adopted throughout the experimental period, and the indoor environment varied naturally with day and night cycles. All reactors were arranged with identical placement positions and light-receiving areas, so environmental disturbances exerted equivalent effects on the three devices. Each module was assembled with transparent acrylic plates, with dimensions of 33 cm × 20 cm × 23 cm (length × width × height), and filled with 10 kg of substrate. Baffles were installed in the first two modules to force water to enter from the bottom and discharge from the upper part, and the final effluent flowed out from the bottom of the third module. Each group consisted of three modules filled with ceramsite, zeolite, and quartz sand in different combinations. Along the water-flow direction, the units of MCW1 were designated as MCW1-1 (quartz sand), MCW1-2 (ceramsite), and MCW1-3 (zeolite); those of MCW2 were MCW2-1 (ceramsite), MCW2-2 (zeolite), and MCW2-3 (quartz sand); those of MCW3 were MCW3-1 (zeolite), MCW3-2 (quartz sand), and MCW3-3 (ceramsite). All three setups shared a single inlet tank, and the inflow rate was regulated by peristaltic pumps. The designed flow rate was 0.02 m^3^/d with a hydraulic retention time (HRT) of 2 days. MCW1, MCW2, and MCW3 represented three distinct substrate-order configurations rather than experimental replicates. Only one independent system was constructed for each configuration (*n* = 1). Repeated water-quality measurements collected during operation were treated as temporal observations within the same system, rather than as independent experimental replicates. Accordingly, comparisons among the three configurations were descriptive and exploratory, and the observed differences were not interpreted as statistically generalizable treatment effects. The influent water quality data and pollutant loading rates are presented in Table 1.

In this experiment, substrates played multiple roles in nitrogen removal, pollutant transformation and elimination, plant growth promotion, and biofilm adhesion [18]. Three substrates were selected: ceramsite, zeolite, and quartz sand (Table 2).

Ceramsite is widely used due to its low cost, high porosity, and good microbial affinity [19]. As a typical aluminosilicate mineral, natural zeolite exhibits excellent adsorption capacity, chemical stability, reactivity, and ion-exchange capability owing to its unique structure and large specific surface area, making it widely applied in wastewater treatment [20]. Quartz sand is a hard, wear-resistant, and chemically stable non-metallic mineral with acceptable permeability; it is eco-friendly, inexpensive, and readily available, thus being one of the most common substrates in constructed wetlands [21]. Combining these materials therefore allowed adsorption, biofilm-mediated transformation, and physical filtration to occur within the same modular system.

The three substrate sequences were arranged cyclically as quartz sand–ceramsite–zeolite (MCW1), ceramsite–zeolite–quartz sand (MCW2), and zeolite–quartz sand–ceramsite (MCW3). Consequently, each substrate occupied the inlet, intermediate, and outlet positions once, while the substrate composition and total mass remained constant among the systems. Because pollutant loading and environmental conditions vary along the flow path, this arrangement enabled a preliminary assessment of how substrate position affected sequential pollutant removal. Zeolite was expected to enhance ammonium retention, ceramsite to support attached microbial growth, and quartz sand to maintain permeability and provide physical filtration. The design was therefore intended to identify a promising substrate sequence for subsequent replicated validation rather than merely compare three arbitrarily selected combinations. All three substrates were purchased from a material company in Gongyi, Zhengzhou.

*Veronica anagallis-aquatica* was collected from surface streams in Linzhi, Xizang Autonomous Region. Notably, the plant existed as a submerged macrophyte at the time of collection and gradually developed into a free-floating macrophyte during subsequent operation, with rhizomes floating in the wastewater rather than rooting into the substrates. Tan [22] compared the domestic wastewater purification performance of *Rumex patientia* and *Veronica anagallis-aquatica*, and the results showed that removal efficiencies of *Veronica anagallis-aquatica* for ammonium nitrogen, COD, and TP all exceeded 80%. In this study, plant height, root length, shoot number, biomass, and tissue nutrient contents were not measured at transplantation or on day 80. Therefore, net hydrophyte growth and direct N or P uptake by plants could not be quantitatively calculated. The nutrient-removal results were consequently interpreted as whole-system removal arising from combined substrate retention, microbial transformation, and possible plant-associated effects, rather than as direct evidence of plant uptake.

### 2.2. Water-Quality Monitoring and Analysis

Domestic wastewater was collected from the office building of Xizang Agricultural and Animal Husbandry University in LinZhi, Xizang Autonomous Region. The detailed determination methods are presented in Table 3. Water samples were filtered through a 0.45 μm membrane filter prior to analysis. Conventional water quality indicators including TN, TP, NH_4_^+^-N and COD were determined in accordance with Monitoring and Analytical Methods for Water and Wastewater (4th Edition).

### 2.3. Microbial Collection and Detection

A total of 30 microbial samples were collected, comprising 3 baseline samples from the unused ceramsite, zeolite, and quartz sand and 27 samples from the nine modules on days 20, 50, and 80. These time points were selected to represent distinct operational stages. Day 20 corresponded to the end of the start-up phase, when ammonium removal had become relatively stable and initial biofilms were expected to have formed. Day 50 represented the established operational stage after prolonged wastewater exposure but before the performance disturbance observed around day 60. Day 80 represented the final stage of the experiment and was selected to characterize microbial communities after the disturbance and subsequent system adaptation or recovery. These sampling points were intended to capture temporal microbial succession rather than exact peaks in microbial biomass, which were not continuously measured in this study. Prevention of cross-contamination between samples: All sampling tools were sterilized at high temperature, dedicated sampling spoons were used for each group of substrates, and aseptic operations were implemented throughout the whole process. The samples were placed in sterilized centrifuge tubes, centrifuged at 3000 r·min^−1^ for 15 min using a high-speed desktop refrigerated centrifuge [23], and stored at −80 °C. All samples were entrusted to Majorbio Bio-Pharm Technology Co., Ltd. (Shanghai, China) for DNA extraction and 16S rRNA gene sequencing. Raw paired-end sequencing reads were quality-controlled using fastp [24] (https://github.com/OpenGene/fastp (accessed on 25 October 2025), version 0.23.4) and merged using FLASH [25] (https://ccb.jhu.edu/software/FLASH/ (accessed on 25 October 2025), version 1.2.11) with the following criteria: Bases with a quality score below 20 at the read tails were trimmed. A sliding window of 50 bp was set, and bases downstream were truncated if the average quality score within the window was below 20. Reads shorter than 50 bp after filtering were discarded, and reads containing more than 5 ambiguous bases (N) were removed. Paired reads were merged into a single sequence based on overlap relationships, with a minimum overlap length of 10 bp. The maximum mismatch ratio allowed in the overlap region of merged sequences was 0.2, and unqualified sequences were filtered out. Samples were distinguished by barcodes and primers at both ends of sequences, and sequence directions were adjusted. The allowed mismatches for barcodes were 0, and the maximum primer mismatches were 2. USEARCH [26,27] (http://drive5.com/usearch/ (accessed on 25 October 2025), version 11) was used to cluster quality-controlled and assembled sequences into operational taxonomic units (OTUs) at a 97% sequence identity threshold, and chimeric sequences were removed. Taxonomic annotation was performed using the RDP Classifier [28] (version 2.11) against the SILVA 138.2/16S bacteria database with a confidence threshold of 70%. Sequences annotated as chloroplasts and mitochondria were removed from all samples before analysis. To minimize the influence of sequencing depth on subsequent analysis, the abundance data of 30 samples were rarefied to 30,554 sequences per sample, yielding a total of 7894 OTUs.

Microbial alpha-diversity indices and beta-diversity were calculated using Mothur (version 1.30.2) and visualized with R software (version 3.3.1). The overall dissimilarity of bacterial communities among samples was evaluated by PCoA based on Bray–Curtis distances combined with ANOSIM/Adonis. The distribution of dominant taxa was visualized using community bar plots and heatmaps. Kruskal–Wallis or Wilcoxon tests were used as exploratory tools to describe differences among microbial samples, and LEfSe analysis was applied to identify discriminative taxa. Because MCW1, MCW2, and MCW3 were single treatment trains rather than replicated experimental treatments, these statistical outputs were interpreted as sample-level or temporal association patterns, not as inferential evidence for generalizable treatment effects among configurations. For functional prediction, 16S rRNA gene data were analyzed using PICRUSt2 [29] (version 2.2.0). All data analyses were performed on the Majorbio Cloud Platform (https://v.majorbio.com (accessed on 25 October 2025)).

### 2.4. Module-Level Apparent Contribution to Pollutant Removal

The apparent contribution of each module to pollutant removal was calculated using a mass-balance approach. For pollutant k (NH_4_^+^-N, TN, TP, or COD), the contribution of module j at sampling time t was calculated as:

(1) $$CR_{j,t}^{k}=\frac{Q_{t\left(C_{j-1,t}^{k}-C_{j,t}^{k}\right)}}{Q_{t}C_{0,t}^{k}}\times 100\%=\left(\frac{C_{j-1,t}^{k}-C_{j,t}^{k}}{C_{0,t}^{k}}\right)\times 100\%$$

where *CRj*,*t*(*k*) is the apparent contribution of module j to the removal of pollutant k at sampling time t; C0,t(k) is the system influent concentration; *Cj* − 1,*t*(*k*) and *Cj*,*t*(*k*) are the influent and effluent concentrations of module j, respectively; and Qt is the flow rate. For the first module, *Cj* − 1,*t*(*k*) is equal to C0,t(k). Because the three modules were connected in series and operated at the same flow rate, Qt was canceled from the equation.

The mean apparent contribution of each module over the monitoring period was calculated as:

(2) $$C{\bar{R}}_{j}^{k}=\left(\frac{1}{n}\right)\sum_{t=1}^{n}CR_{j,t}^{k}$$

where *n* is the number of sampling dates. The sampling-specific contribution was calculated first, followed by arithmetic averaging across the monitoring period. A negative value indicated net pollutant release from a module rather than removal. At each sampling time, the sum of the three module contributions was equal to the overall removal efficiency of the corresponding system. Repeated observations over time were treated as temporal measurements rather than independent experimental replicates.

## 3. Results

### 3.1. Water Quality Parameters

**Figure 2:**
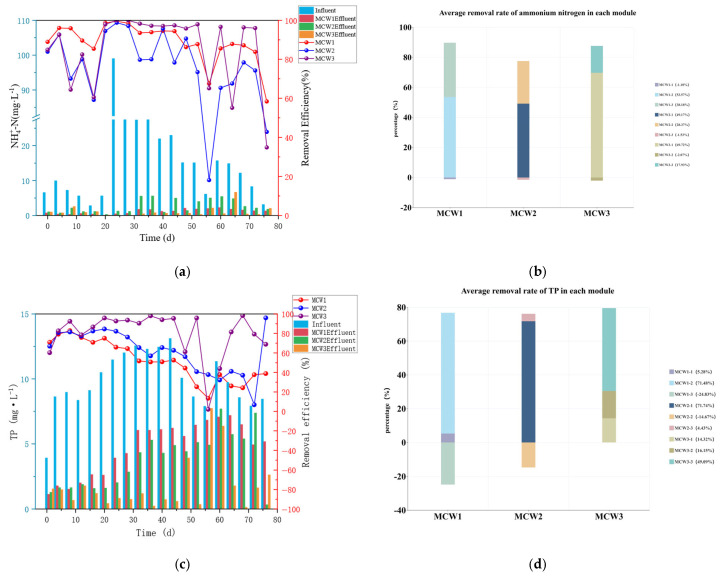
(**a**) Removal efficiency of NH4+-N; (**b**)Average removal rate of NH4+-N in each module; (**c**) Removal efficiency of TP; (**d**) Average removal rate of TP in each module.

**Figure 3:**
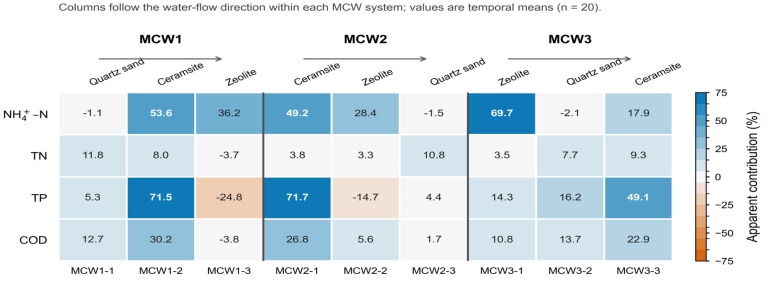
Apparent module contributions to pollutant removal.

The experiment was operated for 80 days, with water quality parameters including NH_4_^+^-N, TN, TP, and COD measured every four days. The influent concentration, effluent concentration, and removal rate of the three systems are shown in Figure 2 and Figure 3.

Overall 80 days, with water quality monitored every 4 days. The results showed configuration-specific differences in pollutant-removal efficiencies among the substrate sequences. MCW3 (zeolite -> quartz sand -> ceramsite) showed the highest observed mean removal efficiencies for TP, TN, and COD among the three configurations. During the late-stage disturbance, its NH_4_^+^-N removal declined and subsequently rebounded, with greater temporal variation than that of MCW1. MCW1 (quartz sand -> ceramsite -> zeolite) showed the highest observed mean NH_4_^+^-N removal efficiency and the lowest TP removal efficiency. MCW2 (ceramsite -> zeolite -> quartz sand) exhibited intermediate pollutant-removal values and temporal fluctuations during operation.

#### 3.1.1. Ammonium Nitrogen (NH_4_^+^-N) Removal

The temporal variations of NH_4_^+^-N removal efficiencies for the three systems are shown in Figure 2a,b and Figure 3. The full-cycle average removal rates of MCW1, MCW2, and MCW3 were 88.6%, 76.01%, and 85.5%, respectively. MCW1 showed the highest observed mean NH_4_^+^-N removal, while MCW3 showed a similar descriptive level.

The operational stages were defined retrospectively according to changes in influent NH_4_^+^-N concentration and removal-performance trajectories rather than as independently imposed loading regimes. Days 0–20 were designated as the start-up phase because the freshly packed substrates were undergoing initial conditioning and the attached microbial communities were still developing. During this period, MCW1 achieved an average NH_4_^+^-N removal efficiency of approximately 91%, whereas MCW2 and MCW3 achieved approximately 77%. The rapid initial removal was consistent with a substantial contribution from physical adsorption by the fresh substrates [30]. However, because substrate adsorption capacity, microbial biomass, and nitrification rates were not independently quantified, its dominance could not be confirmed. Days 20–60 were defined as the established operational phase because MCW1 and MCW3 generally maintained NH_4_^+^-N removal efficiencies above 90% after the initial acclimation period, whereas MCW2 showed a lower mean removal efficiency of 79.2% and greater temporal variability. Sustained removal after prolonged wastewater exposure, together with the detection of nitrifying and other nitrogen-transforming microorganisms, was consistent with an increasing microbial contribution to NH_4_^+^-N transformation. However, because adsorption, reaeration capacity, and individual nitrogen-transformation rates were not independently quantified, nitrification could only be considered a potentially important pathway during this stage rather than conclusively identified as the dominant mechanism. Days 60–80 were defined as the late-stage disturbance phase because influent NH_4_^+^-N increased from 6.12 to 15.70 mg L^−1^ between two adjacent monitoring points, followed by divergent performance responses among the systems. Because this increase was not experimentally imposed, this period was interpreted as a naturally occurring operational disturbance rather than a controlled shock-loading experiment. MCW1 maintained comparatively stable removal during most of this period, indicating greater resistance to the disturbance. In contrast, MCW3 exhibited a marked decline followed by a rapid rebound, suggesting short-term resilience but comparatively weak resistance because of its greater temporal variability. MCW2 maintained a lower overall removal efficiency and did not show consistently superior resistance or recovery.

The apparent contribution of each module to NH_4_^+^-N removal was calculated using Equation (1), and the results are summarized in Figure 2. Zeolite and ceramsite showed positive mean contributions in the tested configurations, ranging from 28.37% to 69.72% and from 17.93% to 53.57%, respectively. In contrast, quartz sand showed small negative mean contributions ranging from −2.07% to −1.10%, indicating net NH_4_^+^-N release at some monitoring points. In MCW3, the inlet zeolite module exhibited an apparent mean contribution of 69.72 ± 33.48% relative to the influent NH_4_^+^-N load across 20 temporal observations. The corresponding contributions of the downstream quartz-sand and ceramsite modules were −2.07 ± 25.39% and 17.93 ± 17.42%, respectively. Thus, the value of 69.72% represents the apparent reduction relative to the influent NH_4_^+^-N load rather than the proportion of total NH_4_^+^-N removed by the complete system. The comparatively high contribution of the inlet zeolite module was consistent with its ammonium ion-exchange capacity [31]. However, because substrate type and module position were not independently replicated, this result may also reflect the higher pollutant loading at the inlet and should be interpreted as a configuration-specific observation.

Mechanistic implications and future optimization: The stepped modular configuration was designed to increase air–water contact during intermodule water transfer and may have promoted passive oxygen transfer. However, dissolved oxygen profiles and oxygen-transfer rates were not measured; therefore, the contribution of the stepped configuration to nitrification could not be determined directly. Intermittent aeration, particularly in the zeolite and ceramsite modules, could be evaluated in future replicated experiments as a potential strategy for treating wastewater with high NH_4_^+^-N concentrations [32].

#### 3.1.2. Total Phosphorus (TP) Removal

The temporal variations of TP removal efficiencies are shown in Figure 2c,d and Figure 3. The full-cycle average removal rates of MCW1, MCW2, and MCW3 were 51.92%, 61.51%, and 79.56%, respectively. MCW3 showed the highest observed TP removal among the three configurations.

Initial phase (days 0–10): All three systems exhibited high TP removal efficiencies during the initial operating period. This pattern may have resulted from rapid phosphate sorption by the fresh substrates and, where Ca- or Al-containing surface sites were available, possible surface complexation or precipitation reactions. Similar phosphorus-retention pathways have been reported for mineral substrates used in constructed wetlands [33]. Mid-term phase (10–52 days): The removal rates of MCW1 and MCW2 gradually decreased, consistent with Zhao [34], who reported that adsorption sites become saturated over long-term operation, leading to declining phosphorus removal capacity. In contrast, the removal rate of MCW3 continued to rise and stabilized above 90%, indicating a favorable configuration-specific TP-removal pattern. Late phase (52–80 days): All three systems showed varying degrees of fluctuation in removal rates, but MCW3 remained above 70%.

Mechanism analysis: Phosphorus removal in constructed wetlands may occur through substrate sorption, chemical precipitation, microbial immobilization, and plant uptake [35]. The cation-rich composition and porous structure of ceramsite may facilitate phosphorus adsorption and provide potential attachment sites for phosphorus-accumulating organisms (PAOs), thereby possibly contributing to phosphorus removal [36]. In this study, all ceramsite-filled modules had higher phosphorus removal contributions than other substrates, especially the final ceramsite module in MCW3, which effectively intercepted phosphorus not removed by preceding modules.

Optimization suggestion: For treating high-phosphorus wastewater, an additional ceramsite advanced treatment unit can be installed at the system outlet. An outlet ceramsite module warrants evaluation in longer, replicated studies.

#### 3.1.3. Total Nitrogen (TN) Removal

**Figure 4:**
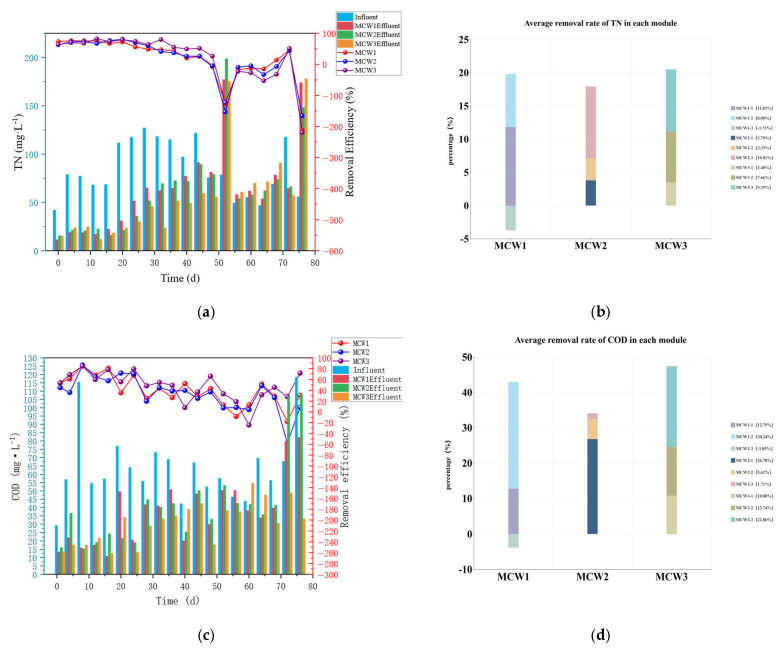
(**a**) Removal efficiency of TN; (**b**)Average removal rate of TN in each module; (**c**) Removal efficiency of COD; (**d**) Average removal rate of COD in each module.

The temporal variations of TN removal efficiencies are shown in Figure 3 and Figure 4a,b. The full-cycle average removal rates of MCW1, MCW2, and MCW3 were only 16.1%, 17.93%, and 20.49%, respectively, with small descriptive differences among the three groups. The overall nitrogen removal performance was unsatisfactory.

Start-up phase (0–20 days): All three systems showed relatively high removal rates, averaging 70–73%, mainly relying on substrate adsorption. Mid-term phase (20–48 days): Removal rates continued to decline and turned to negative removal (effluent TN concentration higher than influent) by day 48. Late phase (48–80 days): Removal rates fluctuated around negative values, indicating the system was in a net nitrogen release state.

The relatively low TN removal efficiency may be attributed to limitations in coupled nitrification and denitrification. Although the stepped configuration was adopted to enhance passive reaeration, dissolved oxygen (DO) was not monitored in this study, so insufficient oxygen supply and its inhibitory effect on nitrification are speculated to be responsible [37]. Carbon source limitation: The low C/N ratio of plateau domestic wastewater resulted in insufficient electron donors for denitrifying bacteria, preventing complete denitrification [38]. Although the device is equipped with a water drop aeration function, dissolved oxygen content may be insufficient to support the growth of nitrifying bacteria. The results revealed a severe shortage of carbon sources in the influent, with the C/N ratio ranging from 0.44 to 2.12 and an average value of only 0.83. Zhang [39] demonstrated that adding bagasse fermentation liquid (C/N = 5) under intermittent aeration conditions could increase TN removal rate to 98.3%.

Future studies could evaluate installing intermittent aeration devices in MCW3, particularly within the zeolite and ceramsite modules. Aeration periods may enhance oxygen transfer and nitrification, whereas non-aeration periods may create relatively oxygen-limited conditions favorable for denitrification. Agricultural residues could also be evaluated as slow-release carbon sources in the modules.

#### 3.1.4. Chemical Oxygen Demand (COD) Removal

As illustrated in Figure 3 and Figure 4c,d, the average COD removal efficiencies of MCW1, MCW2 and MCW3 were 39.14%, 34.11% and 47.40%, respectively. Overall, MCW3 exhibited the highest average COD removal efficiency among the tested configurations, reaching 47.40%. However, this average performance was not interpreted as evidence of superior overall stability or disturbance resistance. The average influent COD concentration throughout the experiment was 63.73 mg/L.

The COD removal efficiency of all three systems gradually declined with prolonged operation. This decline may have been associated with progressive substrate clogging, which can promote hydraulic short-circuiting and dead-zone formation, thereby reducing COD removal [40]. Dissolved oxygen (DO) is recognized as one of the pivotal factors governing COD degradation. Previous studies have demonstrated that tidal flow operation, effluent recirculation and artificial aeration can effectively improve COD removal performance [41].

In this study, the start-up phase showed the highest overall pollutant removal during the operational cycle. Using the apparent module contribution rate defined in the Methods, the ceramsite module exhibited the highest mean COD contribution within each system. The values were 30.24 ± 27.88% for MCW1-2, 26.78 ± 18.01% for MCW2-1, and 22.86 ± 28.43% for MCW3-3 (*n* = 20 sampling events). However, the relatively large SDs indicate considerable temporal variability; therefore, these differences were interpreted descriptively. The relatively high contribution of ceramsite may be associated with its large specific surface area and micro- and mesoporous structure, which may facilitate short-term pollutant adsorption [42]. Over prolonged operation, its porous structure may also provide attachment sites for biofilms and potentially promote the biodegradation of organic pollutants [43].

Accordingly, the current modular configuration requires further optimization of hydraulic flow paths, and regular filler cleaning is suggested to mitigate substrate clogging. Furthermore, installing auxiliary aeration devices in the superior-performing MCW3 group is recommended to elevate DO levels and further enhance COD removal efficiency.

### 3.2. Microbial Community Structure Analysis

A total of 30 microbial samples were collected, including three baseline samples from fresh, unused ceramsite, zeolite, and quartz sand before wastewater introduction and biofilm formation, and 27 operational samples from the nine modules on days 20, 50, and 80. The microbial community characteristics were systematically analyzed from the perspectives of alpha diversity, beta diversity, species composition, and functional prediction.

#### 3.2.1. Alpha Diversity Analysis

**Figure 5:**
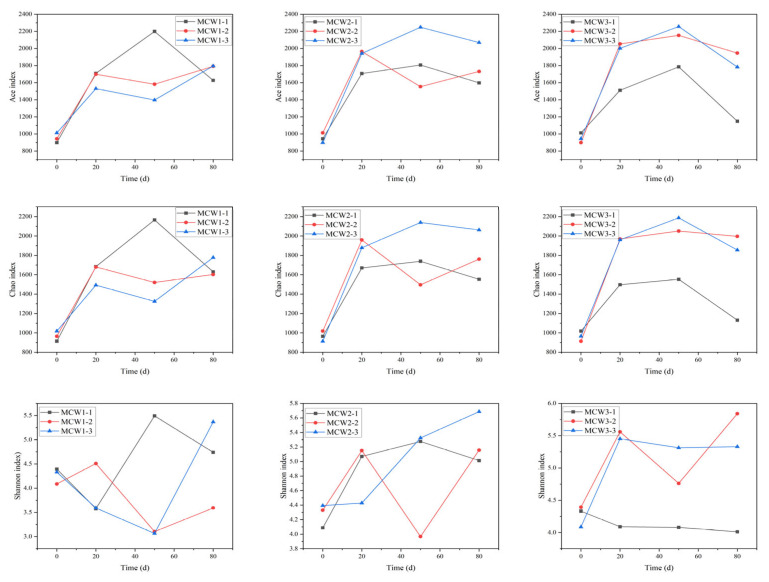
Alpha diversity indices.

Alpha diversity reflects the richness and evenness of microbial communities within a single sample. The variations in ACE index, Chao index, and Shannon index under different substrate combinations and operation periods are shown in Figure 5.

The Coverage index of all samples was above 0.98, indicating sufficient sequencing depth and that the sequencing results suggest adequate sequencing coverage in the samples. Microbial alpha diversity was evaluated using the ACE and Chao1 indices for estimated community richness and the Shannon index for overall diversity, incorporating both richness and evenness.

Species richness indices (ACE, Chao, Sobs). The variation trends of ACE and Chao indices were highly consistent among the three systems: microbial communities acclimated rapidly in the first 20 days of operation, with increased observed species richness; richness reached a peak around day 50 and then slightly decreased.

Descriptive differences in species richness were observed among substrate modules: the Sobs, ACE, and Chao indices of the quartz-sand modules were consistently the highest, followed by the ceramsite modules, whereas the zeolite modules were the lowest. The richness indices of the zeolite modules showed a continuous downward trend with prolonged operation. This pattern suggests that quartz sand might be more conducive to microbial attachment and growth, potentially sustaining higher species richness. Studies have shown that quartz sand provides almost no soluble metal ions or strong adsorption sites, and microbial communities are mainly filtered by environmental factors such as organic matter concentration and dissolved oxygen gradient rather than strong chemical adsorption, thus retaining more species and improving community richness [44].

The Shannon index reflects species evenness and overall microbial diversity. The Shannon index of the quartz-sand modules increased continuously with operation time, indicating improved community diversity. The Shannon index of the ceramsite modules fluctuated gently, indicating moderate community stability, smooth microbial succession, and environmental adaptability. Although ceramsite was not associated with the highest species richness, it may favor the enrichment of certain functional bacteria, potentially contributing to functional diversity and treatment performance [45]. The Shannon index of the zeolite modules decreased in the middle stage of operation, indicating that dominant bacteria gradually occupied a larger proportion of the community and that community structure became simplified. However, when considered alongside the water-quality results, the relatively high NH_4_^+^-N removal in the zeolite modules may have been associated with nitrifying bacterial activity. Because the abundance and activity of these functional bacteria were not quantified, this interpretation remains tentative.

From the overall performance of the three systems, MCW1 exhibited the strongest environmental buffering capacity, with a gentle microbial acclimation process. The community diversity and richness increased synchronously under long-term operation, and it had the best late-stage influent fluctuation resistance, which was consistent with its stable ammonium nitrogen removal effect. MCW2 belonged to a steady-state working condition, with slow microbial community succession, conservative structure, small variation range, and medium diversity maintenance. In MCW3, richness indices increased by day 50 and subsequently declined by day 80, indicating temporal restructuring of the microbial community. However, because microbial biomass, species turnover, and resilience were not directly measured, these patterns cannot establish either superior or inferior ecological stability. The relatively large NH_4_^+^-N removal fluctuations during the late-stage disturbance indicate limited treatment-performance resistance, whereas the subsequent rebound suggests a degree of short-term recovery.

#### 3.2.2. Community Composition Analysis

**Figure 6:**
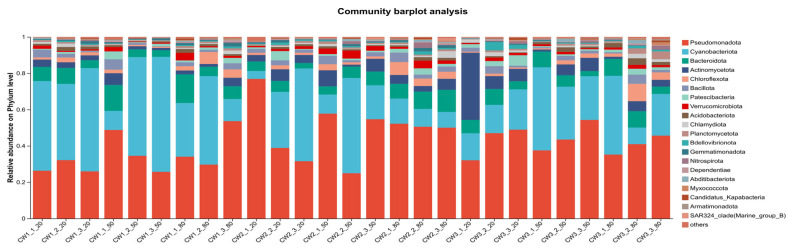
Relative abundance of bacterial community at phylum level.

Microbial community composition is a major factor influencing the water purification function of constructed wetlands. Different substrate types and operation conditions can affect the structural characteristics of microbial communities. In this study, the distribution patterns, taxonomic composition, and literature-reported potential functions of dominant microorganisms under different substrate combinations were analyzed at the phylum and genus levels, and the results are shown in Figure 6.

The composition of microbial communities at the phylum level is shown in Figure 6. The dominant phyla included Pseudomonadota, *Cyanobacteriota*, Bacteroidota, and Actinomycetota, collectively accounting for more than 58% of the total community, with individual relative abundances ranging from 4.61% to 39.51%.

Pseudomonadota exhibited the highest average relative abundance. It remained the dominant phylum (32.06–54.29%) in MCW2 from day 50 to day 80 and in MCW3 throughout the entire operation period (day 0 to day 80). Pseudomonadota comprises metabolically diverse taxa, some of which have been associated with organic-matter degradation and nitrogen transformation in wastewater-treatment systems [46]. This finding is consistent with the results reported by HAO [47], who found that Pseudomonadota exhibited the highest species abundance in plateau environmental experiments, indicating that Pseudomonadota may serve as one of the dominant phyla in plateau wastewater treatment systems. In particular, the early relative abundance of Pseudomonadota in the MCW2-1 (ceramsite) module was nearly 80%, which may reflect that the inlet ceramsite environment is highly suitable for the proliferation of Pseudomonadota. Although its abundance decreased in the later stage, it still accounted for 52.19% of the total community abundance. In MCW3, the relative abundance of Pseudomonadota gradually increased along the flow direction and remained relatively stable, which may reflect that the environmental conditions of MCW3 favored the long-term stable growth of this phylum without intense competitive suppression by dominant photosynthetic taxa. Consequently, a stable community structure dominated by Pseudomonadota and Cyanobacteriota was formed, reflecting the divergent succession patterns of microbial communities under different substrate combinations.

Cyanobacteriota ranked as the second most dominant phylum. As photoautotrophic microorganisms, cyanobacteria rely on light and inorganic nutrients and serve as ubiquitous model organisms for oxygenic photosynthesis, nitrogen fixation, circadian rhythm regulation, symbiosis, and adaptation to harsh environments [48]. Cyanobacteriota was the signature taxon of MCW1; its population expanded continuously from the early stage and eventually became absolutely dominant in the later stage. Combined with the alpha-diversity results, MCW1 showed a lower Shannon index than MCW2 and MCW3. This pattern coincided with the relatively high abundance of Cyanobacteriota and may reflect reduced community evenness associated with the dominance of a limited number of taxa. However, the present data do not establish that Cyanobacteriota enrichment caused the lower microbial diversity. Comparable reductions in microbial diversity have been observed during cyanobacterial bloom outbreaks [49]. In the later operation stage of CW2, Cyanobacteriota was strongly suppressed by Pseudomonadota, indicating that the operational conditions of MCW2 could effectively restrain the proliferation of Cyanobacteria.

Bacteroidota was the third dominant phylum. Members of Bacteroidota are important heterotrophs capable of degrading complex macromolecular organics into small molecular substances, thereby facilitating organic pollutant removal. This phylum also participates in denitrification and contributes to nitrogen pollutant elimination in wastewater [50]. Yue et al. reported that Bacteroidota played a key role in nitrogen removal, achieving average ammonium nitrogen and total nitrogen removal efficiencies of 95.8% and 88.1%, respectively, during system operation [51]. In the MCW1-1 (quartz sand) and MCW3-1 (zeolite) modules, the relative abundance of Bacteroidota increased with operation time, and the abundance in the quartz sand module was higher than that in the zeolite module, demonstrating that quartz sand provided more favorable conditions for the proliferation of Bacteroidota. All three groups presented stable and slight fluctuations without obvious temporal variation or sharp divergence among treatments, suggesting that Bacteroidota possessed strong environmental adaptability and served as a core microbial group maintaining the basic metabolic stability of all three systems.

Actinomycetota was the fourth dominant phylum. Many species within Actinomycetota possess denitrifying capability and can convert nitrate and nitrite into nitrogen gas via denitrification [52]. Actinomycetota showed a relatively high abundance in MCW3 and was identified as a characteristic phylum of this configuration. This pattern may reflect differences in substrate arrangement and local microenvironmental conditions. However, phylum-level relative abundance alone does not demonstrate a more stable or nutritionally complex environment or the colonization of highly functional microbial populations. The ACE and Chao indices suggested broadly comparable richness among the three systems, whereas community structure and evenness differed more clearly. The higher Shannon diversity index of MCW3 was associated with the enrichment of Actinomycetota, but this association should not be interpreted as direct evidence of improved functional stability.

**Figure 7:**
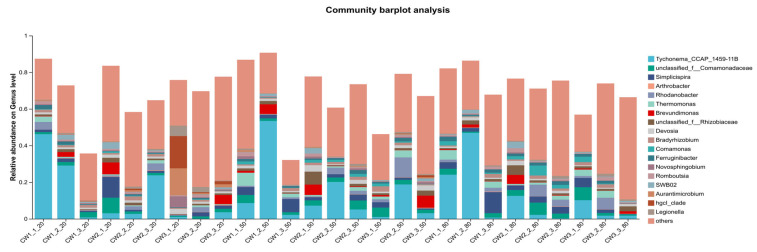
Relative abundance of bacterial communities at genus level.

Figure 7 shows that the top ten bacterial genera were *Tychonema_CCAP_1459-11B*, *Simplicispira*, unclassified_f__Comamonadaceae, *Nitrospira*, *Rhodanobacter*, *Thermomonas*, *Brevundimonas*, unclassified_f__Rhizobiaceae, *Devosia*, and *Bradyrhizobium*.

*Tychonema_CCAP_1459-11B*, a cyanobacterial taxon, was detected mainly in the quartz sand and ceramsite modules, whereas its relative abundance was lower in the zeolite modules. Among the three configurations, MCW3 showed the lowest relative abundance of this taxon. Cyanobacteria may participate in nutrient cycling through photosynthesis and biomass turnover in wetland systems [53]. However, relative abundance alone cannot demonstrate that *Tychonema_CCAP_1459-11B* contributed to oxygen production, CO_2_ fixation, C/N regulation, or nutrient release in the present systems. Similarly, its lower abundance in the zeolite modules cannot be conclusively attributed to competition with heterotrophic or denitrifying bacteria because microbial activity, photosynthetic rates, and interspecific interactions were not directly measured. Therefore, the observed distribution indicates a possible association with substrate type and system configuration, while its ecological function and the mechanisms underlying this pattern require further investigation.

*Simplicispira* has been clearly identified as a partial denitrifying bacterium in constructed wetland research. Together with other nitrifying and denitrifying microorganisms, it mediates multiple nitrogen cycling pathways and enhances TN removal performance [54]. This genus stably existed in all three substrate types and all experimental systems. In the present study, *Simplicispira* was detected across all substrate types and experimental systems. This distribution suggests that it may have participated in nitrogen cycling, but its specific contribution to TN removal was not directly verified.

unclassified_f__Comamonadaceae participates in nitrogen cycling and frequently co-occurs with nitrifying bacteria such as *Nitrospira* [55]. Therefore, the detection of unclassified_f__Comamonadaceae may indicate potential involvement in nitrogen transformation. However, its participation in heterotrophic nitrification, aerobic denitrification, or other denitrification steps cannot be determined from taxonomic composition and relative abundance alone. Confirmation would require functional-gene, transcriptional, or nitrogen-transformation-rate measurements.

*Rhodanobacter* is one of the predominant denitrifying genera. Its relative abundances in ceramsite, zeolite, and quartz sand modules were 0.15%, 2%, and 3.9%, respectively. The proportion in ceramsite modules was notably lower than in the other two substrates. Previous studies have associated temperature, pH, and dissolved oxygen (DO) with the abundance and diversity of *Rhodanobacter* [56], suggesting that the microenvironment formed by ceramsite may be less favorable for the enrichment of this genus.

Previous studies have associated *Thermomonas* with carbon and nitrogen metabolism and identified it as a potential member of denitrifying microbial communities [57]. In the present study, MCW1 showed the highest relative abundance of *Thermomonas*. Although this pattern was consistent with the nitrogen-removal performance of MCW1, relative abundance alone cannot establish that *Thermomonas* mediated denitrification or was responsible for the observed performance. Confirmation would require functional-gene analysis or direct measurement of nitrogen-transformation rates.

Previous research has reported that *Brevundimonas* may promote the growth of *Chlorella sorokiniana* in algae–activated sludge co-cultures, in which substantial nutrient removal was also observed [58]. In this study, *Brevundimonas* was relatively abundant in the ceramsite modules, which also showed the greatest apparent unit contribution to TP removal. This co-occurrence suggests a possible association between *Brevundimonas* and conditions favorable for phosphorus removal, but does not confirm that this genus was directly responsible for phosphorus elimination. Further functional-gene analyses and phosphorus uptake experiments would be required to verify this potential role.

unclassified_f__Rhizobiaceae belongs to the family Rhizobiaceae but cannot be classified into a specific genus. Rhizosphere microorganisms of this family have been associated with nitrogen-cycling processes such as ammonia oxidation, nitrogen fixation, and denitrification [59]. The relatively high abundance in ceramsite modules suggests that the microenvironment provided by ceramsite may be suitable for the growth and colonization of related rhizosphere microorganisms, but the present data do not quantify their contribution to TN removal.

*Devosia* is widely distributed in soils and sediments contaminated with pesticides, petroleum hydrocarbons, mycotoxins [60] (e.g., DON), and chlorinated organic compounds (e.g., HCH), and it is well known for its capability to degrade various toxic organic pollutants [60]. This genus stably colonized all three substrate types and experimental systems and played a certain role in the degradation of toxic organic matter.

*Bradyrhizobium* is predominantly found in soils. It can form root or stem nodules with various leguminous plants, supplies fixed nitrogen to host plants, and sustains ecosystem productivity in nitrogen-poor soils [61].

In summary, although the three modular systems exhibited broadly comparable species richness, their microbial communities followed different temporal succession patterns. MCW1 developed a relatively uneven community characterized by the enrichment of several dominant taxa, whereas MCW2 exhibited comparatively moderate temporal variation in community composition. MCW3 maintained relatively high diversity at some sampling points but underwent pronounced compositional changes, particularly during late-stage influent fluctuation. Therefore, the diversity observed in MCW3 should not be interpreted as evidence of greater temporal stability. These patterns suggest that substrate sequence and operational conditions may jointly influence microbial community assembly, although the functional roles of specific taxa require further validation.

### 3.3. Correlation Analysis

#### 3.3.1. Correlation Heatmap

**Figure 8:**
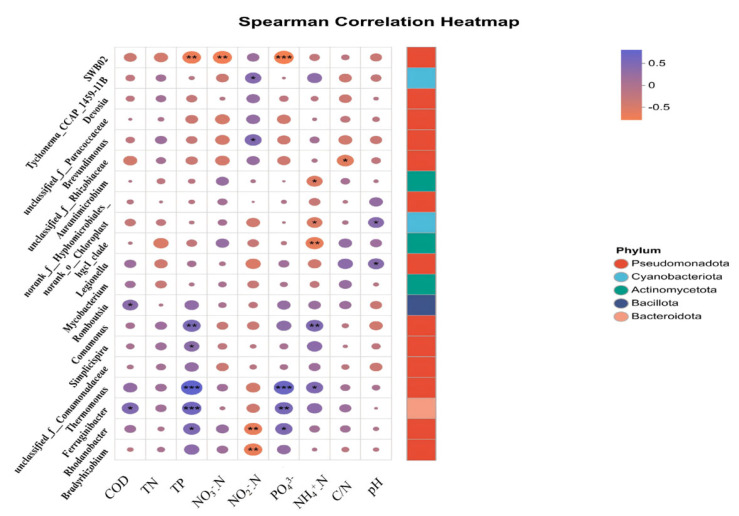
Correlation heatmap (* *p* ≤ 0.05, ** *p* ≤ 0.01, and *** *p* ≤ 0.001).

Figure 8 Spearman correlations between dominant microbial genera and water-quality variables. Circle color and size indicate correlation direction and strength, respectively. Asterisks denote *p*-value thresholds (*p* ≤ 0.05, *p* ≤ 0.01, and *p* ≤ 0.001), and the right-hand color strip indicates phylum affiliation. As shown in Figure 8, *SWB02* was negatively correlated with TP, NO_3_^−^-N, and PO_4_^3−^. Although *SWB02*-related taxa have previously been associated with nitrite oxidation, these correlations do not demonstrate that *SWB02* oxidized NO_3_^−^-N or that NO_3_^−^-N accumulation inhibited nitrification in the present systems. The observed relationships may also reflect shared responses to environmental conditions or indirect microbial interactions.

*Tychonema_CCAP_1459-11B* showed a positive correlation with NO_2_^−^-N. Although nitrogen-fixation potential has been reported for some cyanobacterial taxa, this correlation does not verify nitrogen fixation by *Tychonema_CCAP_1459-11B*. Confirmation would require measurements of nitrogen-fixation genes or activity.

unclassified_f__Rhizobiaceae was negatively correlated with the C/N ratio. Members of Rhizobiaceae have previously been associated with organic-matter degradation and nitrogen transformation. However, the function of this unclassified family-level taxon cannot be determined from its relative abundance and correlation with C/N alone.

*hgcI_clade* exhibited a distinct negative correlation with NH_4_^+^-N. Although this taxon is frequently detected in oligotrophic aquatic environments, the correlation does not demonstrate that it performed denitrification or directly removed ammonium. The relationship may instead reflect its environmental preferences or indirect interactions with other microorganisms.

Comamonas was positively correlated with TP and NH_4_^+^-N. Previous strain-level genomic and physiological studies demonstrated that some Comamonas strains can perform denitrification under carbon-deficient conditions [62]. However, the potential function of Comamonas in the present systems was inferred solely from previous literature and taxonomic annotation. No taxon-specific functional genes or denitrification rates were measured. Moreover, its positive correlations with TP and NH_4_^+^-N may reflect enrichment under nutrient-rich conditions rather than direct pollutant removal.

*Thermomonas* showed positive correlations with TP and PO_4_^3−^. Previous research associated *Thermomonas* with carbon, nitrogen, and energy metabolism in an integrated microalgae pond–constructed wetland system [57]. Therefore, its potential involvement in nutrient transformation was inferred from previous literature rather than directly demonstrated in this study. The observed correlations alone cannot establish nitrogen or phosphorus removal, adaptation to plateau conditions, or functional complementarity with *Comamonas*.

*Ferruginibacter* was positively correlated with TP and PO_4_^3−^. Previous constructed wetland research reported the enrichment of *Ferruginibacter* in iron-amended systems and suggested its potential association with iron transformation and nitrogen cycling [63]. Accordingly, its possible involvement in iron-related nutrient cycling was inferred from previous studies. However, the present correlations provide no direct evidence that *Ferruginibacter* mediated phosphorus removal through iron cycling.

*Rhodanobacter* was positively correlated with TP and PO_4_^3−^, but negatively correlated with NO_2_^−^-N. Previous constructed wetland studies identified *Rhodanobacter* as a member of denitrifying bacterial communities whose abundance may respond to environmental conditions [56]. Thus, its potential denitrification role was inferred from previous literature. The correlations observed here do not verify sulfur-dependent autotrophic denitrification, adaptation to low-carbon conditions, or direct phosphorus removal.

#### 3.3.2. Mantel Test and Environmental Correlation Analysis

**Figure 9:**
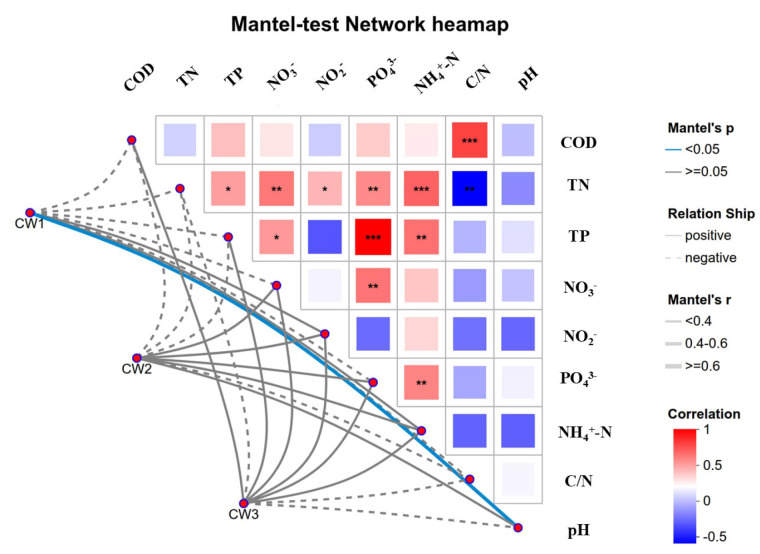
Mantel test analysis plot (* 0.01 < *p* ≤ 0.05, ** 0.001 < *p* ≤ 0.01, and *** *p* ≤ 0.001).

Figure 9 Mantel-test network heatmap showing relationships between microbial community composition and water-quality variables. The heatmap represents Spearman correlations among environmental variables. Red and blue indicate positive and negative correlations, respectively, and color intensity represents correlation strength. Asterisks indicate significance (* 0.01 < *p* ≤ 0.05, ** 0.001 < *p* ≤ 0.01, and *** *p* ≤ 0.001). Network edges represent Mantel correlations between the genus-level microbial community matrices of CW1, CW2, and CW3 and individual environmental-variable distance matrices. Blue and gray edges indicate Mantel-test *p* < 0.05 and *p* ≥ 0.05, respectively. Solid and dashed edges indicate positive and negative correlations, respectively. Edge width represents |Mantel’s *r*|: <0.4, 0.4–0.6, and ≥0.6.

The Mantel test was used to evaluate the relationships between microbial community composition and water-quality variables. The microbial matrix comprised genus-level abundance data from the OTU_Taxon_Depth table and was analyzed separately for CW1, CW2, and CW3. Bray–Curtis dissimilarities were calculated for the microbial community data. The environmental matrix comprised COD, TN, TP, NO_3_^−^-N, NO_2_^−^-N, PO_4_^3−^, NH_4_^+^-N, C/N, and pH values from the corresponding samples, for which Euclidean distances were calculated. Mantel’s *r* and *p* values were used to evaluate the relationships between microbial community dissimilarity and individual environmental variables. Pairwise relationships among environmental variables were evaluated using Spearman’s correlation.

The Mantel test and Spearman correlation results in Figure 9 describe the statistical associations among water quality indicators in the constructed wetland system and their association patterns across different wetland units (MCW1, MCW2, and MCW3). These results should be interpreted as patterns of covariance rather than evidence of direct regulation, causal control, or purification mechanism.

Positive correlations were mainly observed between organic pollutant indicators and nitrogen-phosphorus nutrient indicators. COD was positively correlated with NH_4_^+^-N (*p* < 0.001), indicating that these two variables tended to vary in the same direction across the sampled conditions. TN also showed positive correlations with TP, NO_3_^−^-N, NO_2_^−^-N, PO_4_^3−^, and NH_4_^+^-N, suggesting close co-variation among total nitrogen, inorganic nitrogen species, and phosphorus-related indicators in the dataset.

TN showed a strong negative correlation with the C/N ratio, which was the most pronounced negative association among the indicator pairs. TP was also negatively correlated with NO_3_^−^-N, NO_2_^−^-N, NH_4_^+^-N, and C/N. However, these relationships should be interpreted cautiously because the C/N ratio mathematically includes nitrogen-related terms. Therefore, part of the observed correlation between C/N and nitrogen indicators may arise from mathematical coupling rather than independent ecological or biochemical relationships.

pH was negatively correlated with several indicators, including TN, TP, and NH_4_^+^-N. The apparent strength of these negative associations increased from MCW1 to MCW3. This pattern indicates that pH covaried with the measured water quality indicators across wetland units, but it does not by itself demonstrate that pH directly regulated pollutant removal or system performance.

For the network associations between wetland units and water quality indicators, MCW1 showed stronger positive associations with COD, TN, and TP than the other units. This suggests that variations in these conventional pollutant indicators were more closely aligned with the MCW1 sample pattern. In contrast, the associations of MCW2 and MCW3 with most water quality indicators were weaker or more frequently negative. The negative association between MCW3 and pH was particularly evident. Overall, these results indicate that the three wetland units differed in their correlation structures with water quality indicators. However, the Mantel and Spearman analyses alone cannot identify the underlying processes responsible for these differences, and further experimental or mechanistic evidence would be required to infer purification pathways or regulatory effects.

#### 3.3.3. Single-Factor Correlation Network Diagram

**Figure 10:**
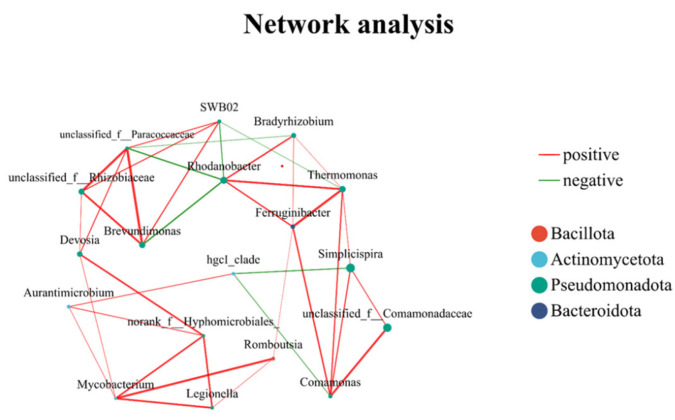
Single-factor correlation network diagram.

Figure 10 Microbial co-occurrence network in the modular constructed wetlands. Node color and size represent phylum affiliation and relative abundance, respectively. Red and green edges indicate positive and negative correlations, while edge thickness represents correlation strength. As shown in Figure 10, positive correlations were observed between unclassified_f__Rhizobiaceae and unclassified_f__Paracoccaceae, *SWB02*, and *Brevundimonas*. *Thermomonas* was positively correlated with *Rhodanobacter*, *Ferruginibacter*, and *Comamonas*, whereas *Comamonas* was positively correlated with unclassified_f__Comamonadaceae and *Ferruginibacter*. These relationships indicate that the relative abundances of these taxa covaried across samples.

Previous studies cited above have confirmed that some of these taxa are involved in nitrogen transformation, organic matter degradation, or iron-related biogeochemical processes. However, the present correlation network does not demonstrate direct cooperation, cross-feeding, or sequential metabolic relationships among these taxa. In particular, it cannot establish that Rhizobiaceae supplied fixed nitrogen, *SWB02* provided carbon substrates, or the correlated taxa formed a complete nitrogen-cycling pathway. Positive correlations may also result from shared environmental preferences, indirect interactions, or the compositional nature of relative-abundance data. Edge thickness represents correlation strength rather than functional importance or interaction intensity. Therefore, the predominance of positive correlations should not be interpreted as evidence of a stable microbial community or well-developed metabolic chains. Verification of these potential interactions would require functional-gene, transcriptional, metabolite, or isotope-tracing analyses.

### 3.4. Discussion of Correlation Analysis Results

Correlation analyses revealed associations among microbial taxa, water-quality variables, and environmental conditions. *SWB02*, hgcI_clade, *Tychonema_CCAP_1459-11B*, Comamonas, *Thermomonas*, Ferruginibacter, and *Rhodanobacter* were associated with different nitrogen- or phosphorus-related variables, although these relationships indicate statistical covariation rather than directly verified biochemical functions. Among the environmental variables, TN was positively correlated with TP, NO_3_^−^-N, NO_2_^−^-N, PO_4_^3−^, and NH_4_^+^-N but negatively correlated with C/N, whereas COD was positively correlated with C/N. The Mantel test network further showed that associations between microbial community dissimilarity and environmental variables varied among MCW1, MCW2, and MCW3. Although positive taxon-taxon correlations predominated in the microbial network, they may reflect shared environmental preferences, indirect associations, or compositional effects rather than microbial cooperation, complete metabolic pathways, or community stability. Therefore, functional-gene, transcriptional, metabolite, or isotope-tracing analyses are required to verify the ecological functions and interactions inferred from these correlation patterns.

## 4. Discussion

The results indicate that the central context of this study is not a replicated comparison of treatment technologies, but an exploratory evaluation of how substrate sequence shaped pollutant removal and microbial succession in modular constructed wetlands under a plateau low-temperature habitat. Substrate sequence redistributed treatment functions among modules rather than producing one universally superior configuration. MCW1 showed the highest observed mean NH_4_^+^-N removal, whereas MCW3 showed the highest observed mean TP and COD removal. TN removal remained limited in all three systems. Therefore, the main contribution of the study is the identification of configuration-specific performance patterns and microbial responses under the tested high-altitude operating conditions.

The plateau habitat is important for interpreting the organic-pollution results. Low temperature usually slows microbial growth, enzyme activity, biofilm development, and organic-matter mineralization in constructed wetlands. Despite this constraint, MCW3 achieved an observed mean COD removal of 47.40%, and the ceramsite modules showed relatively high apparent COD contributions. This result suggests that organic-pollution reduction was still possible under the tested low-temperature conditions. The likely explanation is a combination of physical retention, adsorption within porous media, and biofilm-mediated mineralization by microbial assemblages that acclimated during the 80-day operation. However, BOD was not measured separately, and oxygen profiles, respiration rates, and organic-matter mineralization kinetics were not quantified. The observed COD reduction should therefore be interpreted as evidence of partial organic-load attenuation rather than as direct proof of complete biological mineralization.

The low-temperature setting also helps explain the divergence between NH_4_^+^-N and TN removal. Zeolite-containing modules, especially when placed at the inlet, showed favorable NH_4_^+^-N reduction, which is consistent with ammonium ion exchange and possible subsequent nitrification in biofilms. In contrast, TN removal remained low because complete nitrogen removal requires both nitrification and denitrification. The influent C/N ratio averaged only 0.83, indicating a shortage of electron donors for heterotrophic denitrification. Low temperature could further restrict denitrifier activity. Thus, the favorable NH_4_^+^-N removal and weak TN removal are not contradictory; they indicate that ammonium retention or oxidation occurred more readily than complete nitrogen conversion to gaseous end products.

The interpretation of nitrogen and phosphorus removal must also account for the absence of hydrophyte growth data. In constructed wetlands, N and P removal can be directly related to plant uptake when plant biomass and tissue nutrient contents increase during operation. In the present study, plant height, root length, shoot number, biomass, and tissue N or P concentrations were not recorded. Therefore, the removal of NH_4_^+^-N, TN, and TP cannot be quantitatively attributed to *Veronica anagallis-aquatica* uptake. The observed nutrient removal more conservatively reflects whole-system processes, including substrate sorption, chemical precipitation, microbial immobilization or transformation, and possible plant-associated rhizosphere effects. Because the plants developed floating rhizomes rather than rooted growth in the substrates, plant uptake may have contributed to nutrient removal, but its magnitude remains unverified.

Microbial richness did not correspond directly to pollutant-removal performance. Quartz-sand modules generally exhibited higher Sobs, ACE, and Chao1 values, whereas zeolite modules showed lower richness but favorable NH_4_^+^-N removal. Thus, greater richness does not necessarily indicate stronger microbial attachment, functional stability, or treatment efficiency. MCW3 maintained relatively high alpha diversity at some sampling points but underwent pronounced temporal restructuring between days 50 and 80. Moreover, the functions of detected taxa were inferred from previous literature and taxonomic annotation. The Spearman, Mantel, and network analyses identified association patterns but did not demonstrate microbial functions, cooperation, or causal relationships.

These findings provide preliminary evidence that modular substrate sequencing may support pollutant-specific optimization of constructed wetlands in high-altitude settlements. Nevertheless, each configuration contained only one treatment train, and only three of the six possible substrate sequences were evaluated. The experiment was also limited to an 80-day indoor summer period without continuous dissolved-oxygen, hydraulic, plant-growth, BOD, or substrate-chemistry measurements. Future studies should employ replicated systems, longer seasonal operation, additional substrate configurations, complete influent pollution-load profiling, direct plant-growth and tissue-nutrient measurements, and process-level microbial assays.

## 5. Conclusions

This pilot-scale study evaluated three modular constructed wetland configurations over 80 days. The systems exhibited different pollutant-removal and microbial succession patterns. However, because each configuration was represented by a single treatment train, the comparisons should be interpreted as configuration-specific observations.

1. Water quality purification performance

MCW1 achieved the highest average NH_4_^+^-N removal efficiency of 88.6%, whereas MCW3 showed the highest average TP and COD removal efficiencies of 79.56% and 47.40%, respectively. Apparent unit-contribution analysis supported a substantial contribution of the ceramsite module to TP removal under the tested configurations. However, these results do not establish that ceramsite is indispensable in all plateau constructed wetlands. TN removal remained limited in all three systems. Intermittent aeration may potentially improve NH_4_^+^-N oxidation and COD degradation, whereas external carbon addition may enhance denitrification. These strategies were not tested and should therefore be evaluated in future optimization studies.

2. Microbial diversity

Microbial richness increased during the early operational stage, reached relatively high levels around the middle stage, and subsequently declined. Quartz-sand modules generally exhibited higher richness indices, followed by ceramsite and zeolite modules. Despite its comparatively lower microbial diversity, zeolite maintained favorable NH_4_^+^-N removal. This pattern may reflect its ion-exchange capacity or the selective enrichment of certain microbial taxa, but it does not demonstrate reliance on a small number of highly efficient functional bacteria. MCW3 exhibited relatively high alpha diversity at some sampling points but also showed pronounced temporal changes during late-stage influent fluctuation. Thus, high diversity in MCW3 should not be interpreted as evidence of superior temporal or functional stability.

3. Microbial community composition

At the phylum level, Pseudomonadota, Cyanobacteriota, Bacteroidota, and Actinomycetota were the dominant phyla, collectively accounting for 58% of the total community. The relative abundances of each bacterial phylum differed across configurations and operational stages. Several detected genera have previously been associated with nitrification, denitrification, phosphorus cycling, or organic-matter degradation. However, these potential functions were inferred from taxonomic annotation and previous literature rather than directly verified in this study. The three configurations followed different succession patterns: MCW1 exhibited greater dominance by several taxa, MCW2 showed comparatively moderate temporal variation, and MCW3 maintained relatively high diversity at some stages but underwent more pronounced compositional changes during late-stage influent fluctuation.

4. Correlation analysis

Correlation analyses identified associations among microbial taxa, water-quality variables, and community-composition patterns. These correlations indicate statistical covariation rather than directly verified microbial functions or causal relationships. Although positive taxon–taxon correlations predominated in the network, they do not demonstrate microbial cooperation, complete metabolic pathways, or community stability. The Mantel-test results indicated configuration-specific associations between microbial community dissimilarity and environmental variables, but they did not establish that individual variables directly controlled treatment performance. Functional-gene, transcriptional, metabolite, and process-rate measurements are required to verify the proposed ecological functions and microbial interactions.

## Figures and Tables

**Table 1:** Influent water quality and pollutant loading rates.

Water Quality Parameters	Concentration Range (mg·L^−1^)	Mean ± Standard Deviation	Mass Loading Rate (g·d^−1^)	Areal Loading Rate (g·m^−2^·d^−1^)	Volumetric Loading Rate (g·m^−3^·d^−1^)
COD	29.24–118.43	63.73 ± 21.53	1.275	6.44	31.86
NH_4_^+^-N	2.81–99.00	18.38 ± 21.32	0.368	1.86	9.19
TN	41.92–127.12	84.49 ± 28.54	1.690	8.53	42.24
TP	3.92–13.12	9.81 ± 2.22	0.196	0.99	4.90

**Table 2:** Parameters of the substrates.

Item	Ceramsite	Zeolite	Quartz Sand
Particle size (mm)	3–5	6–8	2–4
Apparent density (g/cm^3^)	1.65	1.22	2.65
Bulk density (g/cm^3^)	0.99	1.20	1.65
Porosity (%)	40	32–48	33–51

**Table 3:** Analytical methods.

Water Quality Parameter	Analytical Method	Instrument Model	Manufacturer
COD	Potassium dichromate method	6B-3000A	Jiangsu Shengaohua Environmental Protection Technology Co., Ltd., Changzhou, China
TN	Ultraviolet spectrophotometry with potassium persulfate digestion	6B-3000A	
NH_4_^+^-N	Nessler’s reagent spectrophotometry	6B-3000A	
TP	Molybdenum-antimony anti-spectrophotometry	6B-3000A	

## Data Availability

The water quality data supporting this paper were obtained from the Xizang Civil Engineering, Water Conservancy and Electric Power Engineering Technology Research Center, and the microbial data were obtained after detection by Majorbio Bio-Pharm Technology Co., Ltd. The original contributions presented in this study are included in the article. Further inquiries can be directed to the corresponding author.
